# Supplementary material for: Hormonal Blood Pressure Regulation during General Anesthesia Using a Standardized Propofol Dosage in Children and Adolescents Seems Not to Be Affected by Body Weight
Source: J Clin Med. 2020 Jul 6;9(7):2129. doi: 10.3390/jcm9072129 (PMC7408938; doi:10.3390/jcm9072129)
Supplement: Supplementary file 1 [file jcm-09-02129-s001.pdf]

Journal of
Clinical Medicine

MDPI

Article

Supplement

Table S1: Preoperative hormone levels (median [Q1-Q3]; [1]Mann-Whitney-U-test

	n	group „regular weight“	n	group „overweight“	p-value[1]
**Renin** (mU/l)	40	16.35 [8.40-30.35]	17	20.70 [15.20-43.00]	0.160
**Angiotensin II** (pmol/l – adjusted)	40	6.47 [5.61-12.76]	17	7,77 [6.62-11.44]	0.979
**Aldosterone** (pmol/l)	40	215.00 [137.85-396.90]	17	172.00 [136.60-239.40]	0.542
**Copeptin** (pmol/l)	40	4.01 [3.29-5.65]	16	3.98 [3.09-5.42]	0.562
**Epinephrine** (pg/ml)	36	44.00 [27.50-70.50]	15	27.00 [19.00-50.50]	0.102
**Norepinephrine** (pg/ml)	37	177.00 [137.00-289.00]	15	219.00 [162.00-326.00]	0.327

Journal of
Clinical Medicine

Table S2: absolute change of plasma-hormone-concentration 15 min after induction of anesthesia (median [Q1-Q3]; [1]Mann-Whitney-U-test)

**Δ after 15 min**	**n**	**group „regular weight“**	**n**	**group „overweight“**	**p-value[1]**
**Renin** (mU/l)	39	23.6 [8.0-59.2]	17	46.1 [21.4-94.8]	0.216
**Angiotensin II** (pmol/l – adjusted)	40	15.5 [7.0-28.0]	17	19.3 [13.8-31.1]	0.191
**Aldosterone** (pmol/l)	40	-17.0 [-66.1-132.7]	17	66.0 [-31.0-151.0]	0.513
**Copeptin** (pmol/l)	40	-0.9 [-1.9- -0.4]	16	-0.6 [-1.4- -0.4]	0.269
**Epinephrine** (pg/ml)	36	-28.0 [-48.5- -18.0]	15	-19.0 [-38.5- -14.0]	0.247
**Norepinephrine** (pg/ml)	35	-128.0 [-182.0- -89.0]	15	-152.0 [-258.0- -107.0]	0.342

Journal of
Clinical Medicine

MDPI

Table S3: absolute change of plasma-hormone-concentration 45 min after induction of anesthesia (median [Q1-Q3]; [1]Mann-Whitney-U-test)

Δ after 45 min	n	group „regular weight“	n	group „overweight“	p-value[1]
**Renin** (mU/l)	40	34.4 [6.3-57.9]	17	41.3 [22.4-109.5]	0.125
**Angiotensin II** (pmol/l – adjusted)	40	16.4 [5.0-28.5]	17	22.7 [17.7-31.2]	0.078
**Aldosterone** (pmol/l)	40	35.4 [-55.7-217.4]	17	119.0 [-5.2-300.3]	0.337
**Copeptin** (pmol/l)	40	-0.6 [-1.7-0.7]	16	1.0 [-1.1-2.0}	0.182
			14		
**Epinephrine** (pg/ml)	31	-32.0 [-43.5- -19.0]	14	-11.0 [-35.0-4.0]	**0.031**
**Norepinephrine** (pg/ml)	35	-100.0 [-162.5- -42.0]	15	-133.0 [-226.5- -65.5]	0.478

© 2020 by the authors; licensee MDPI, Basel, Switzerland. This article is an open access article distributed under the terms and conditions of the Creative Commons Attribution (CC BY) license (http://creativecommons.org/licenses/by/4.0/).
